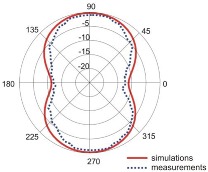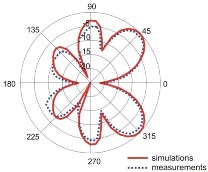# Many-Objective Automated Optimization of a Four-Band Antenna for Multiband Wireless Sensor Networks

**DOI:** 10.3390/s18103309

**Published:** 2018-10-02

**Authors:** Łukasz Januszkiewicz, Paolo Di Barba, Łukasz Jopek, Sławomir Hausman

**Affiliations:** 1 Institute of Electronics, Lodz University of Technology, 90-924 Łódź, Poland; lukasz.jopek@p.lodz.pl (Ł.J.); slawomir.hausman@p.lodz.pl (S.H.); 2 Department of Electrical, Computer and Biomedical Engineering, University of Pavia, I-27100 Pavia, Italy; paolo.dibarba@unipv.it

**Keywords:** multi-band antenna, wireless sensor networks, Paretian optimization, evolutionary computing, 5G networks

## Abstract

This paper describes a new design and an optimization framework for a four-band antenna to be used in wireless sensor networks. The antenna is designed to operate effectively in two open frequency bands (ISM—Industrial, Scientific, Medical), 2.4 GHz and 5.8 GHz, as well as in two bands allocated for the fifth-generation (5G) cellular networks, 0.7 GHz and 3.5 GHz. Our initial design was developed using the trial and error approach, modifying a circular disc monopole antenna widely used in ultra wideband (UWB) systems. This initial design covered the three upper bands, but impedance matching within the 700 MHz band was unsatisfactory. The antenna performance was then improved significantly using an optimization algorithm that applies a bi-objective fully-Paretian approach to its nine-parameter geometry. The optimization criteria were impedance matching and radiation efficiency. The final design exhibits good impedance matching in all four desired bands with the Voltage Standing Wave Ratio (VSWR) value below 2 and radiation efficiency of 88%. The simulated antenna performance was verified experimentally.

## 1. Introduction

Wireless sensor networks have become increasingly common in recent years, as they support an ever-expanding array of applications, in agriculture [1], marine environment monitoring [2], ambient assisted living [3], industry [4], healthcare [5], and beyond. Often, such networks do not have a dedicated licensed frequency band, but operate in the existing radio environment. They may make use of open bands (e.g., ISM—Industrial, Scientific, Medical), which do not require licenses [6]. Due to limitations on transmit power, open bands are especially suitable when the operating range of each node is not very large. In complex application scenarios which include large distances or multiple users, cellular wireless systems can be used for data transfer from the sensor network to the core network. This technology can be combined with low power, short distance systems such as ZigBee or Bluetooth, which operate in the ISM band [7]. Multiple transmission standards are still available in the ISM band.

In the near future, the fifth generation of wireless communication systems (5G) will also become more widely used. This set of wireless technologies combines low-power, low-data rate transmission with high-data rate, low latency links. Numerous wireless sensor networks that utilize low power nodes transmit low amounts of data at low transmission speeds. These will benefit from the development of the special low power transmission protocols that 5G will make available in the 700 MHz band [8]. At the same time, 5G systems will enable high data rates in the 3.5 GHz band, making this band attractive for designers of nodes which aggregate measurement data from wireless sensor networks [9].

The wideband and multiband antennas have received a lot of research attention in the last few years. Numerous multiband and multi-system transmission schemes which require such antennas have been put forward. In [10], a novel wide-band microstrip antenna for wideband applications is proposed which consists of a square radiating patch and a partial ground plane. Since the antenna bandwidth covers the range of 2.1711 to 4.0531 GHz it can be used for Wireless Local Area Network (WLAN), WiMAX (Worldwide Interoperability for Microwave Access—a family of wireless communication standards) and Long Term Evolution (LTE) systems. The antenna presented in [11] is based on a microstrip design as well. It is a modified rectangular patch antenna with the U-shaped defected ground structure (DGS) unit and two parasitic elements (open-loop-ring resonators). It can operate in 4 frequency bands from 4.4 GHz up to 10 GHz and is suitable for applications in WLAN and WiMAX systems. The design of multiband antenna whose geometry is controlled by many parameters can be significantly improved with computer optimization technique. In [12] the multiband antenna is presented with the radiator which uses the inverted “F” geometry. The successful utilization of the two-stage optimization algorithm resulted with the design that covers two bands: 824–960 MHz and 1710–2170 MHz. In this paper, we describe the design and procedure of optimization for a four-band antenna to be used in wireless sensor networks. The novel antenna we present is able to operate effectively in two open bands, 2.4 GHz and 5.8 GHz, as well as in two bands that are licensed and allocated for 5G wireless systems, 0.7 GHz and 3.5 GHz. The bands listed in Table 1 are likely to be used in wireless sensor networks. There are already many small low power wireless transmission modules designed for ISM bands (bands 2 and 4 in Table 1). The bands allocated for 5G systems (bands 1 and 3 in Table 1) will use low power protocols developed for the Internet of Things. It will also be possible to achieve backhaul transmission in the 3.5 GHz band with 5G systems.

**Figure 1:**
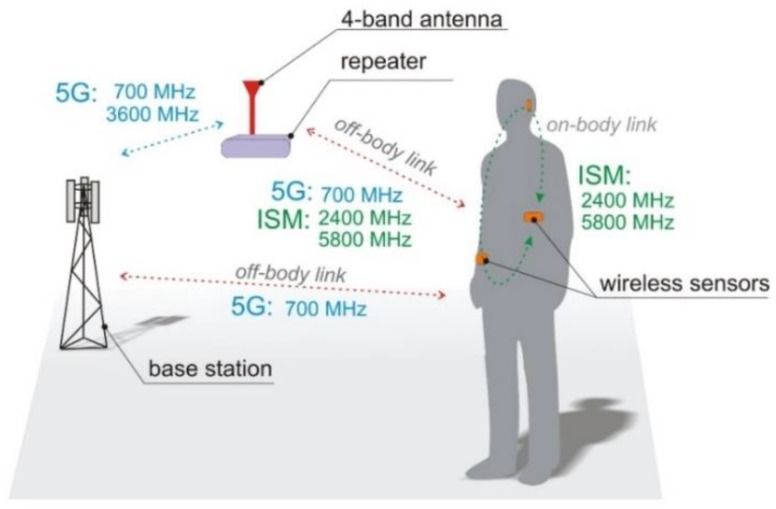
Transmission scenario in wireless sensor network.

The potential data transmission scenario in which the four-band antenna will be used is presented in Figure 1. It is assumed that the antenna can be connected to a four-band transceiver which will communicate with a wireless body area network in bands 1, 2 and 4 of Table 1. ISM bands (2 and 4) are widely used for wireless body area networks due to the availability of low power transceivers. Nodes in wireless body area networks will soon also operate in band 1, and be compatible with the 5G Internet of Things. Band 3 can be utilized for high-speed transmission in 5G systems. These assumptions justify the selection of the bands covered by the proposed antenna. 

**Figure 2:**
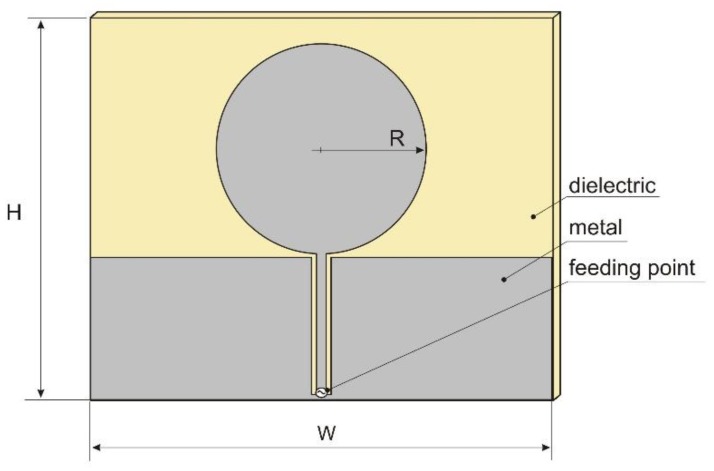
The coplanar waveguide (CPW)-fed circular disc monopole antenna.

The initial antenna considered for this application was a coplanar waveguide (CPW) fed circular disc monopole antenna, illustrated in Figure 2. A detailed description of this antenna can be found in [13]. The antenna was designed for ultra wideband (UWB) systems, covering the frequency range from 2.27 GHz to 12 GHz. The original dimensions were: radius R = 25 mm and width W = 90 mm. The antenna was designed on 1.6 mm thick dielectric substrate with relative permittivity equal to 3.

**Figure 3:**
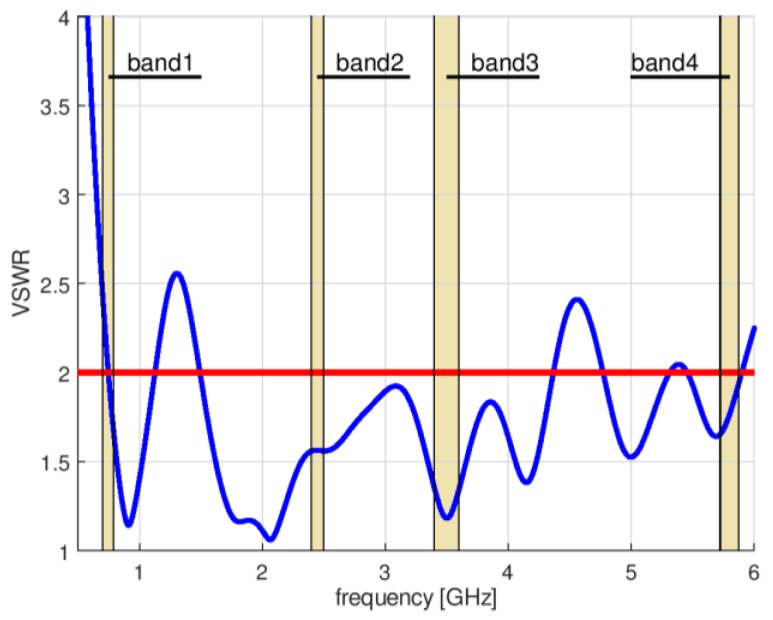
Impedance matching of the CPW-fed circular disc monopole antenna.

To obtain UWB antenna operation in the lower frequency range of 703 MHz (band 1), it was necessary to redesign the original antenna. For this purpose, a numerical model of a circular disc monopole antenna was created in Remcom XFdtd software [14]. This program uses the Finite Difference Time Domain method (FDTD) to simulate electromagnetic devices [15]. The lower frequency of operation was achieved by increasing the radius of the circular part to R = 45 mm. The width of the antenna was in this case equal to W = 200 mm and height was increased to H = 165 mm for the same dielectric substrate. The impedance matching of this antenna is presented in Figure 3. The desired bandwidth was achieved with the Voltage Standing Wave Ratio VSWR < 2.5, which is not a satisfactory result. Further increasing the antenna radius resulted in improved matching for the lower frequency of interest, but performance for the upper band deteriorated. Moreover, the dimensions of the antenna (200 mm × 165 mm) and its surface area equal to 0.033 m^2^ made this design rather impractical for applications in wireless sensor networks, if they should have small nodes. 

**Figure 4:**
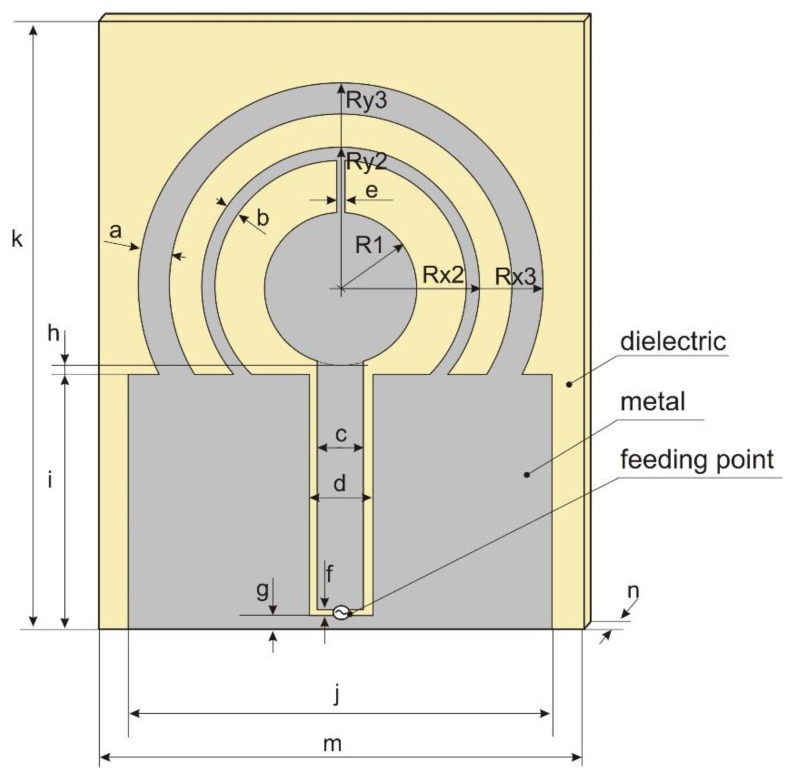
Four-band antenna layout and dimensions.

**Figure 5:**
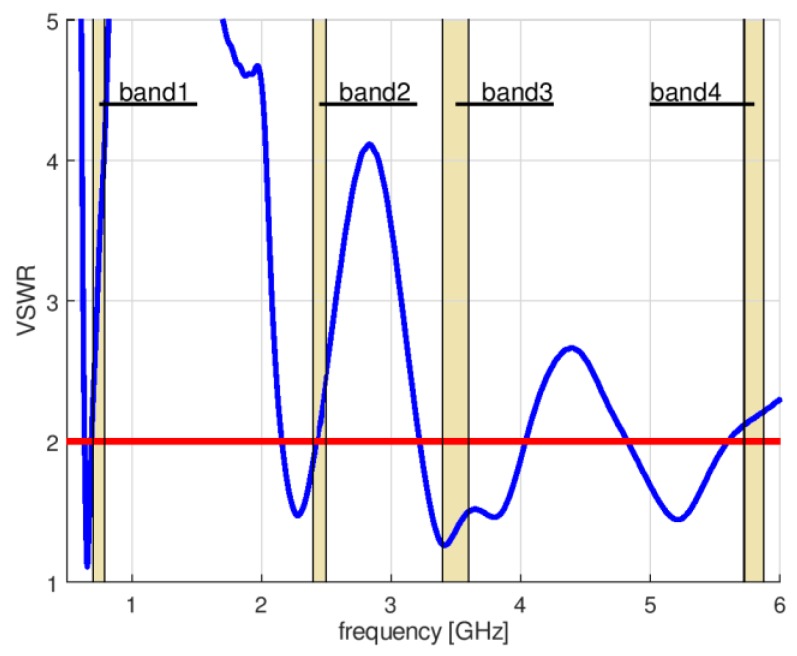
Impedance matching of initial design of four-band antenna.

To overcome the limitations of the circular disc monopole antenna, a new design was proposed and developed for a four-band antenna. The antenna geometry is presented in Figure 4. It is fed by a coplanar waveguide and consists of one full internal circle and two external rings (circular or elliptical). The design parameters are listed in Table 2. Initial values were obtained by trial and error using Remcom XFdtd software ver. 7.6.0.2. The original design covers the two middle bands with a satisfactory level of impedance matching (VSWR < 2.3), but the impedance matching in the bands 1 and 4 was insufficient and the VSWR in band 1 was less than 4.5. The impedance matching of the initial design is presented in Figure 5. The performance of this antenna depends strongly on the geometrical configuration. Of the 18 design parameters listed in Table 2, the first 9 have a strong influence on impedance matching. 

The large number of parameters made further improvement of the proposed antenna using trial and error very difficult, because of the tremendous computational effort required to simulate many possible combinations of parameter values. We therefore employed an automated optimization algorithm. The application of optimization algorithms can significantly improve antenna performance [16,17,18,19,20]. Nine design parameters were included in the optimization process, while the other nine remained fixed. The goal was to improve impedance matching in the four bands without degrading the radiation properties of the antenna. The impedance properties of the antenna were evaluated against the VSWR parameter calculated for the reference impedance of 50 Ω. The radiation properties of the antenna were assessed in terms of radiation efficiency (RE), i.e., the ratio of radiated power to the input power at the feed point. 

## 2. Multi-Objective Optimization Algorithm

**Figure 6:**
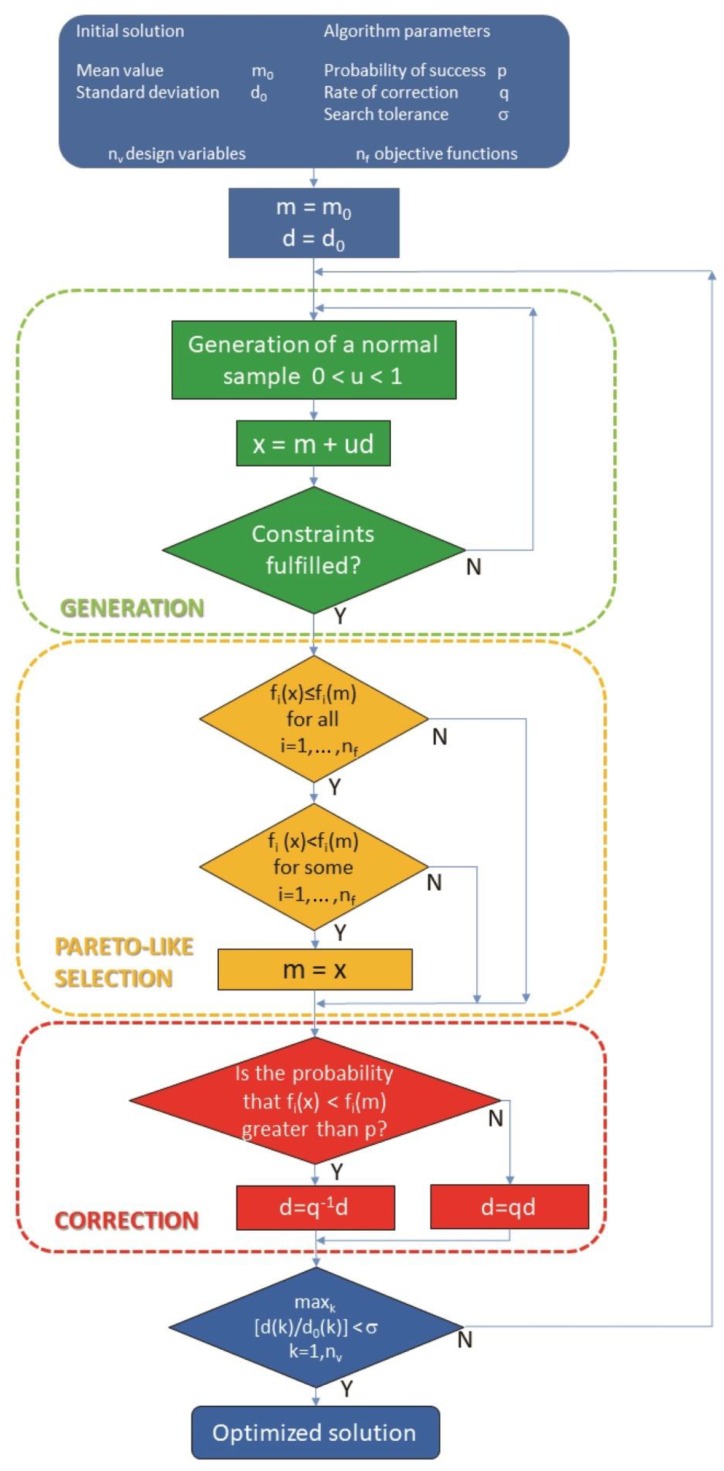
Flow chart of P-Estra algorithm.

The optimization of multiband antennas with respect to impedance matching and radiation efficiency raises a bi-objective optimization problem. Moreover, the multiple evaluation of both objective functions based on full-wave electromagnetic simulations makes the optimization procedure computationally costly. To help with these difficulties, several years ago one of the authors developed a simple yet effective algorithm based on a multi-objective (1+1) (i.e., one parent is used to create one offspring) evolution strategy (P-EStra), which is presented in [21,22]. The flow-chart of the algorithm is shown in Figure 6. It is implemented in such a way that a new design vector *x* (offspring solution) is generated from the current design vector *m* (parent solution) according to (1)

(1) x=m+du

where *d* is the standard deviation associated with *m*, while u∈[0,1] is a normally distributed perturbation. Provided it fulfills the problem constraints, solution *x* is accepted if *x* dominates the current design vector *m* (parent solution), i.e., given *n_f_* objective functions

$$f_{i}\left(x\right)\leq f_{i}\left(m\right)\;\mathrm{for}\;\mathrm{each}\;\mathrm{value}\;\mathrm{of}\;i\in [1,n_{f}]\;$$

$$f_{k}\left(x\right)<f_{k}\left(m\right)\;\mathrm{for}\;\mathrm{at}\;\mathrm{least}\;a\;\mathrm{value}\;\mathrm{of}\;k\in [1,n_{f}]$$

according to the Pareto optimality criterion [22]. Otherwise, vector *m* is retained. 

Vector *d*, which drives the search, is in turn updated in the following way: given the correction rate q∈(0,1), in the *k*-th iteration, either

(2a) $$d_{k+1}=q^{-1}d_{k}$$

or

(2b) $$d_{k+1}=qd_{k}$$

is set to force a larger (2a) or smaller (2b) standard deviation of the Gaussian distribution associated with *x* in the next iteration. The choice of *d_k_*_+1_ value depends on the probability of successful iteration, i.e., the rate of success in improving the objectives. The solution vector *x* and the standard-deviation vector *d* are both subject to mutation. In a basic (1+1) implementation, the operator of Pareto-like selection allows the best individual, whether parent or offspring, to survive to the next generation. In this way, given an initial solution there is a non-zero probability that the optimization trajectory will lead eventually to a solution belonging to the Pareto optimal front of non-dominated solutions, i.e., the best compromises trading off objective functions. The basic computational cost *c* of the algorithm can be estimated as

(3) $$c\approx c_{0}\cdot n_{i}\cdot n_{p}\cdot n_{f}$$

where *c*_0_ is the hardware-dependent time necessary to run a single FDTD analysis, *n_i_* is the number of convergence iterations for a prescribed search accuracy, *n_p_* is the number of individuals, and *n_f_* is the number of objectives.

More generally, starting from an initial population of individuals spanning the feasible region of the problem, P-EStra produces a final population which approximates the Pareto optimal front. In this case, however, the three major operators (generation, selection, correction) have to be implemented in parallel. The main advantage of this method is the reduced computational cost in terms of algorithm complexity, since there is no need either to sort the current population into Pareto sets in each iteration or to process an external archive of non-dominated individuals.

In summary, P-EStra exploits:

- competition between parent and offspring in the evolution of an individual. This competition is ruled by the Pareto criterion, stimulating convergence to the front within the dominance cone associated with the initial solution;
- diversity among individuals that are processed in parallel. In particular, the number of individuals does not vary during the process and therefore the Pareto front is approximated by a number of points determined a priori. Moreover, no solution is discarded during the process, ensuring full computational efficiency.

The algorithm implies that the final point is closer to the Pareto front than the initial point. However, convergence to the front is proved from the numerical standpoint only. Finally, there is no limitation on the number of objectives the algorithm is able to process, at least in principle, and this is a potential advantage for high-dimensionality problems. A drawback of the algorithm is the lack of a rule forcing the spread of non-dominated solutions in the case of a multi-individual strategy. As a consequence, non-dominated solutions might form clusters which are disseminated along the Pareto optimal front. 

## 3. Application of Pareto Algorithm to Four-Band Antenna Design

The P-EStra optimization algorithm presented above was used to optimize our four-band antenna for multiband wireless sensor networks. The algorithm was implemented in Matlab. The goal of the optimization was to improve antenna performance in terms of two objective function components: impedance matching and radiation efficiency. The two components were evaluated from numerical simulations performed using the Remcom XFdtd full-wave simulation program, in which the antenna geometry model was created automatically in each iteration of the optimization loop using new geometry parameter values. The values (9 of the 18 design parameters listed in Table 2) were generated for each iteration made by the P-EStra algorithm. In XFdtd, five subsequent simulations were performed for each optimization step: one with broadband excitation to obtain the VSWR in all the considered bands and four simulations with harmonic excitation in each of the four middle frequencies (see Table 1) to obtain the radiation efficiency for each band. One goal was to improve the impedance matching of the antenna in the four bands, which corresponded to minimizing the largest value of VSWR. The other goal was to maximize antenna radiation efficiency in the bands. Formally, this optimization problem can be stated as follows:

We define:

*g*: design vector (geometric parameters defining the multi-band antenna shape)
Ω_g_: set of admissible values
B_1_: band at 700 MHz
B_2_: band at 2.4 GHz
B_3_: band at 3.5 GHz
B_4_: band at 5.8 GHz
VSWR: voltage standing wave ratio
RE_1_: radiation efficiency for band 1
RE_2_: radiation efficiency for band 2
RE_3_: radiation efficiency for band 3
RE_4_: radiation efficiency for band 4
RE: minimum radiation efficiency of bands 1–4

Starting from a feasible solution g_0_ within Ω_g_, the following f_1_ objective is to be minimized:

(4) $$f_{1}\left(g\right)=\underset{f\in B_{1234}}{\sup}\left|\mathrm{VSWR}\left(g,x,f\right)\right|,{\;\mathrm{where}\;B}_{1234}=\cup_{k=1}^{4}B_{k}\;,\;g\in \Omega_{g}$$

and, simultaneously, the following f_2_ objective is to be maximized:

(5) $$f_{2}\left(g\right)=\left[\underset{f\in B_{1234}}{\inf}\left|\mathrm{RE}\left(g,x,f\right)\right|\right]\;,{\;\mathrm{where}\;B}_{1234}=\cup_{k=1}^{4}B_{k}\;,\;g\in \Omega_{g}$$

**Figure 7:**
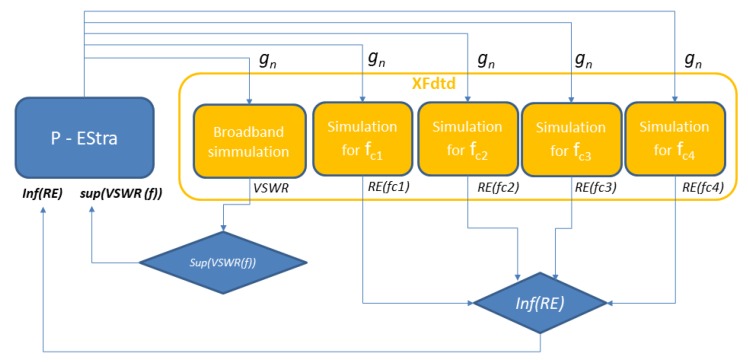
Flowchart of optimization loop combining the P-EStra optimization algorithm and XFdtd simulation program.

The P-EStra algorithm makes the doublet (f_1_,f_2_) evolve from the guess solution *g*_0_ to convergence, keeping f_1_ and f_2_ as individual objectives. The flowchart of the optimization procedure is presented in Figure 7.

## 4. Results of Antenna Optimization with the P-EStra Algorithm

The initial set of design parameter values for our four-band antenna was selected by trial and error. This provided the starting point for antenna optimization using the P-EStra algorithm. The corresponding design parameters are given in Table 2. The initial value for the VSWR component of the objective function was VSWR_start_ = 4.5 and the radiation efficiency component was RE_start_ = 78%. The constraints in the optimization process were geometry-oriented, allowing only for sets of design variable values that preserved the assumed geometry of the antenna without self-intersections or overlapping sections. This required the radius of the external ellipse to be greater than the radius of the internal ellipse, the width of the slot to be greater than the width of the internal strip and so on. The optimization process required 54 iterations to satisfy the automatic stopping condition. The condition relies on the ratios of the standard deviation within the current iteration $d_{k}$ to the initial standard deviation iteration *d*0_k_ for each *k*-th optimization variable. The deviation is normalized across all the variables. The process stops when $\underset{k}{sup}\left[d_{k}/d0_{k}\right]<s$, where *s* is search tolerance. This corresponds to the situation when the current search region is sufficiently small for all variables. In this study, guided by our experience, we assumed $s=10^{-2}$.

**Figure 8:**
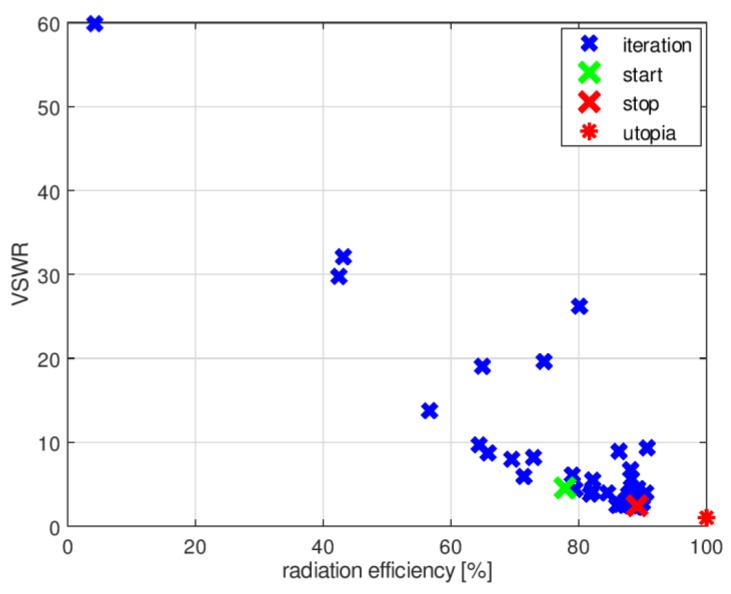
History of optimization process from initial point (see Table 1) in objective function space.

**Figure 9:**
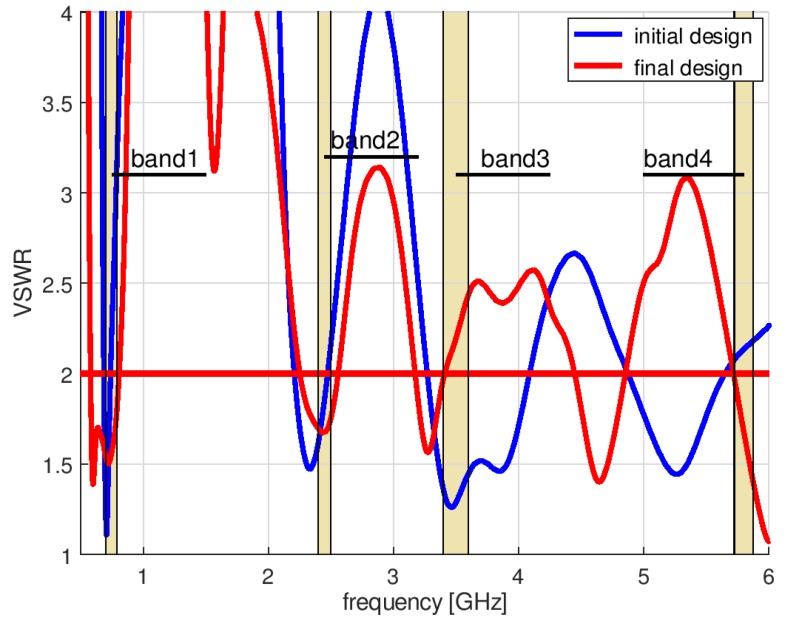
Impedance matching of initial design and following optimization with P-EStra.

The history of the optimization process from the initial point (given in Table 2) in the objective function space is presented in Figure 8, where the utopia solution (the best combination of the objective function components) is marked by a red asterisk. The objective function components for the best solution identified by the algorithm were VSWR_stop_ = 2.43, RE_stop_ = 89%. Figure 9 compares the impedance matching of the initial design with that following optimization with P-EStra. 

**Figure 10:**
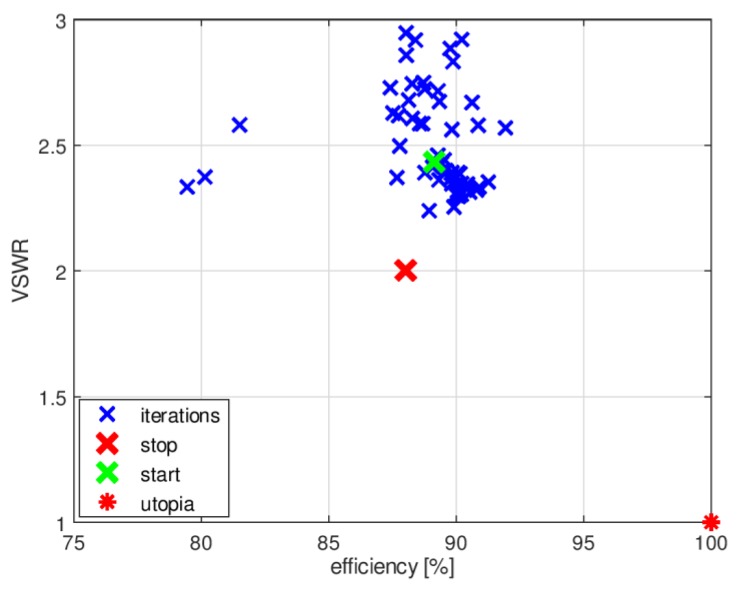
History of the optimization process with VSWR value constraints, presented in the objective function space.

To improve the impedance matching of the antenna in band 3, another run of the optimization algorithm was performed. In this case, the constraints were based on both the geometrical conditions and the objective function values. The constraints were considered as violated for a given set of design variables when the geometry was self-overlapping and when the VSWR value obtained for the proposed geometry was greater than 3. To test the second condition, it was necessary to run XFdtd, which increased the computational burden. Figure 10 shows the history of the optimization process with VSWR limited constraints in the objective function space. The starting point was the same as final point in the previous optimization run.

**Figure 11:**
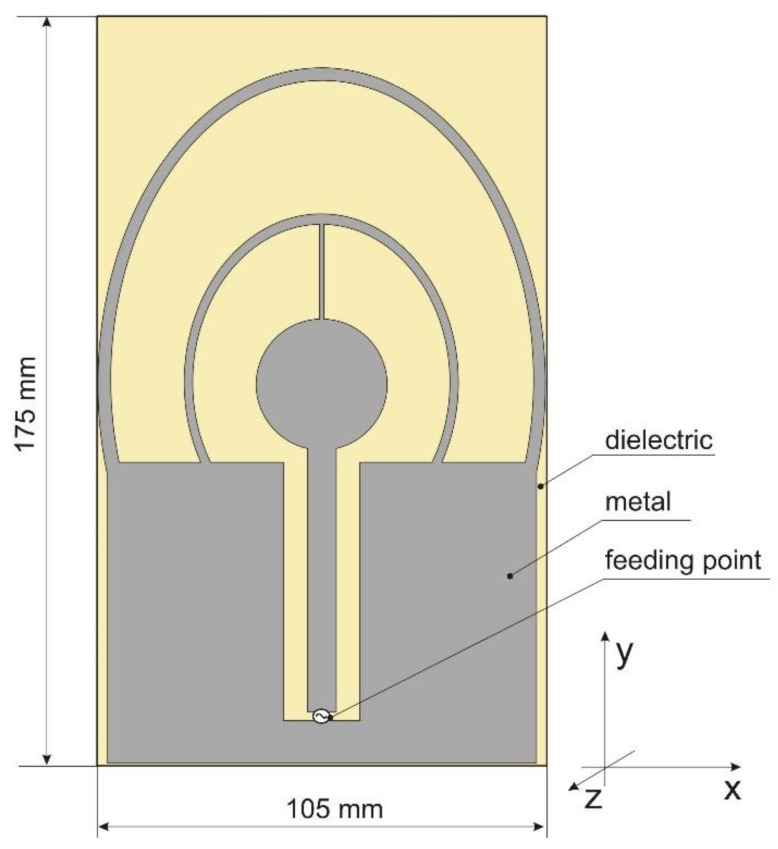
Geometry of optimized antenna.

**Figure 12:**
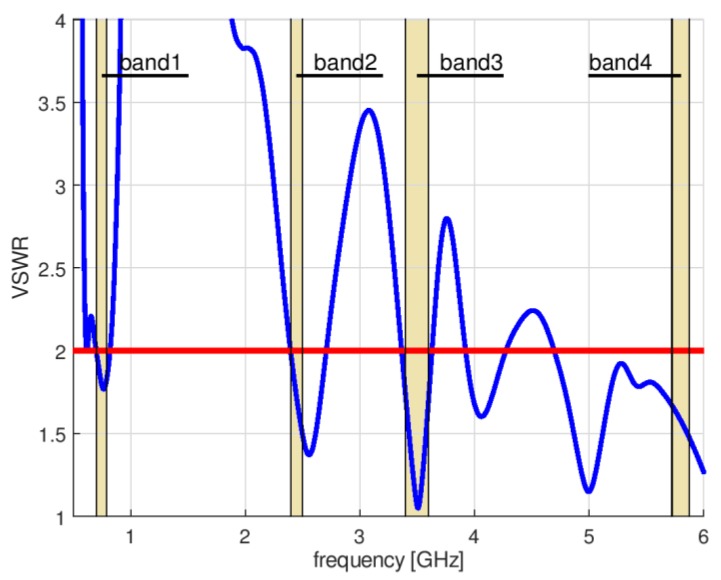
Impedance matching of optimized antenna.

In this case, the optimization algorithm needed 56 iterations to converge. Due to the need to estimate the value of VSWR for each set of design parameter values proposed by P-Estra, 24 more simulations were required using XFdtd. The objective function components for the best solution identified by algorithm were VSWR_stop_ = 2, RE_stop_ = 88%. Table 3 presents the set of design parameters of the optimized antenna design and Figure 11 the corresponding antenna geometry. Figure 12 shows the impedance matching of the antenna optimized using P-EStra. 

**Figure 13:**
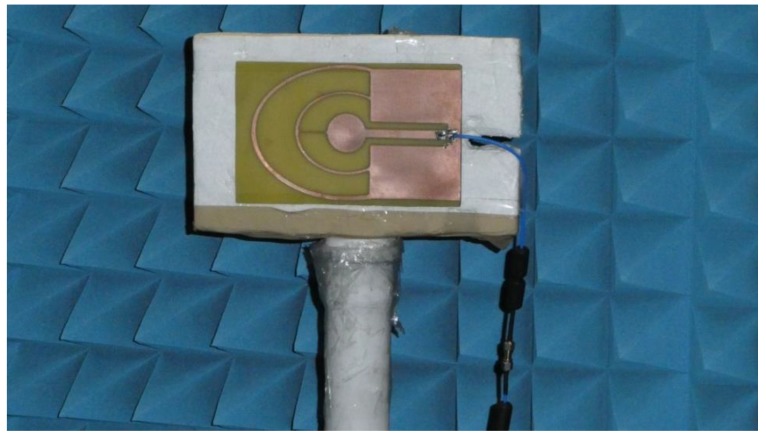
Prototype of four-band antenna in unechoic chamber.

**Figure 14:**
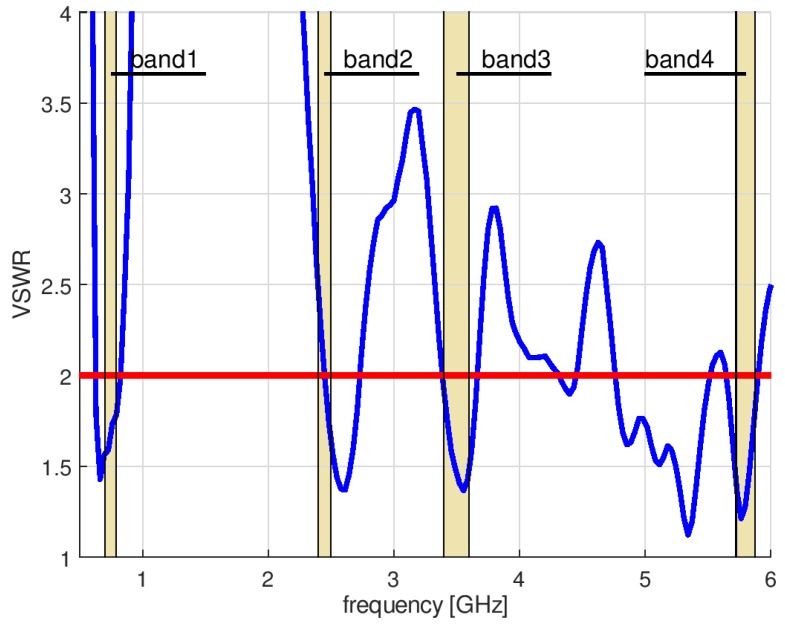
Impedance matching of prototype antenna.

The impedance matching of the optimized antenna was verified experimentally using a prototype. The prototype antenna, fabricated on 1.5 mm thick FR4 substrate (ε = 4, tgδ = 0.01—these values are provided by the substrate manufacturer), is presented in Figure 13. The impedance matching of the prototype antenna was measured using a Rohde & Schwarz ZVB 14 vector network analyzer in an anechoic chamber. Because the prototype antenna was fed by a coaxial probe, the calibration plane was moved to the end of the coaxial cable. The impedance matching of the prototype is presented in Figure 14. The results obtained by means of measurements differ from the simulation results, but the character of VSWR parameter variation is similar. The greatest differences can be observed in band 2, where the maximum value for VSWR was 2.35 versus 2. In the other bands, the maximum VSWR was less than 2. Minor differences between the simulated impedance matching and the measurement results followed from the influence of the feeding of the prototype antenna which used a coaxial cable soldered directly to the printed antenna. In the numerical model of the antenna, the feeding source was connected directly between the internal rectangle and the outer rectangular part. The pieces of solder on the prototype antenna introduced additional volume of conductor detuning the antenna. Also, the limited precision of the prototype antenna geometry, which was fabricated using etching technology might introduce the small discrepancy between the results of simulations and measurements. The dielectric properties of the substrate used for the prototype may also have been different from those assumed during the optimization process.

## 5. Electromagnetic Characterization of the Optimized Antenna

The radiation properties of the antenna in different frequency bands depend on the electric current distribution along the antenna radiator. Analysis of this distribution was performed using Remcom XFdtd software. The antenna was fed with a harmonic signal of the middle frequency of each band (see Table 1). The results are presented in Table 4, in terms of surface current obtained for an antenna radiator surface normalized to 10 A/m. For band 1, the highest current density is on the feeding line edges and on the inner ellipse, while for higher bands it is on the feed line and on the inner circular part.

The fact that current distribution on the antenna surface varies with frequency results in a radiation pattern that also depends on frequency. The radiation patterns of the antenna are presented in Table 5. The spatial orientation of the radiation patterns corresponds to the axis presented in Figure 11. The polarization of the antenna is linear, parallel to the plane of the antenna radiator. For band 1, the antenna exhibits an omnidirectional (with ca. −2 dB variation) radiation pattern in the plane perpendicular to the antenna axis of symmetry (the *z-x* plane) with the maximum gain of 1.3 dBi. For this band, the antenna provides the transceiver with a quasi-circular coverage, which is a desirable feature in mobile wireless systems in which the relative spatial placement of other terminals is not fixed. The distribution of antenna gain in the vertical plane (*z-y*) with a null in the direction of the antenna axis of symmetry is typical of antennas for which currents flow predominantly in a vertically oriented conductor (dipole, monopole, UWB monopole antenna etc.).

For higher frequency bands, the antenna dimensions are comparable or greater than the wavelength. This results with the current distribution on the antenna surface which exhibits multiple local maxima. This results in greater variation of the antenna gain in the *z-y* plane, where side lobes are visible. Also in the *z-x* plane, the gain differs from the perfectly omnidirectional distribution within the range of 7 dB while the maximum gain increases up to 7.2 dBi. These radiation properties are suitable for the assumed communication scenario in which the antenna is a node component in a multiband wireless sensor network because the antenna gain in the *z-y* plane that is assumed to be in the horizontal plane is always greater than −4 dBi which is acceptable for multiband antennas. In the case of the indoor scenario, the local minima in the radiation pattern that are visible for higher bands are acceptable because of multipath propagation which compensates for the influence of the minima.

## 6. Discussion

The lowest frequency of operation of our four-band antenna is 0.703 GHz and the highest 5.875 GHz, which yields an 8.3:1 ratio. Such high ratios are difficult to achieve, especially for antennas which should be smaller than half of the largest wavelength. Our antenna is able to operate in the prescribed frequency bands with good impedance matching (VSWR ≤ 2) and radiation efficiency of 88%. It is also relatively small in size. The largest dimension of the four-band antenna is 175 mm, which is only 0.4 of the longest wavelength considered (in free space). The full dimensions of the antenna are 175 mm × 105 mm with a surface area of 0.018 m^2^. This is significantly smaller than the typical UWB antenna covering a similar frequency band we designed mainly for size comparison, the dimensions of which were 200 mm × 165 mm with a surface area of 0.033 m^2^. Reducing the antenna surface by 55% enables a reduction in antenna mass, which is another desirable feature. Both the four-band antenna and the UWB antenna were designed using the same FR4 substrate, with a thickness of 1.5 mm.

## 7. Conclusions

In this paper, we have presented a novel design for a four-band antenna to be used for multiband wireless sensor networks. The antenna is designed to operate effectively in two open frequency bands (ISM—Industrial, Scientific, Medical), 2.4 GHz and 5.8 GHz, as well as in two bands allocated for the fifth-generation (5G) cellular networks, 0.7 GHz and 3.5 GHz. We have demonstrated an effective method for bi-objective multi-parameter optimization of the antenna geometry, using the P-EStra algorithm. This algorithm was able to improve the complex geometry of our initial multiband antenna, which in the studied case was controlled by nine design parameters. We used a two-dimensional objective function and fully Paretian approach to optimize the antenna with respect to two design criteria (VSWR and radiation efficiency). These criteria each have an important influence on antenna performance. There is no inherent limitation that prevents our optimization methodology from including additional dimensions of the objective function.

Finally, we verified the performance of our optimized four-band antenna design experimentally. Comparison of measurement results with simulations for impedance matching and radiation patterns showed good agreement in all four frequency bands.

## Figures and Tables

**Table 1:** Frequency bands considered for wireless sensor network node.

Band Number	Description	Lower Frequency *f*_l_ (MHz)	Middle Frequency *f*_c_ (MHz)	Upper Frequency *f*_u_ (MHz)
1	5G, IoT	703	740	788
2	ISM	2400	2450	2500
3	5G	3400	3500	3600
4	ISM	5725	5800	5875

**Table 2:** Antenna dimensions—initial values.

	Number	Symbol	Physical Meaning	Initial Value (mm)
Design variables	1	R_1_	radius of smallest circle	18
	2	R_x2_	radius of external ellipse in x direction	46
	3	R_y2_	radius of external ellipse in y direction	46
	4	R_x3_	radius of internal ellipse in x direction	33
	5	R_y3_	radius of internal ellipse in y direction	33
	6	A	thickness of external ellipse	4.5
	7	B	thickness of internal ellipse	3
	8	C	internal rectangle width	10
	9	D	width of slot	15
Constant values parameters	10	E	width of interconnecting rectangle	1
	11	F	width of horizontal slot in feeding point	2
	12	G	height of metal layer at feeding point	10
	13	H	elevation of circular part above rectangle	3
	14	I	height of metal layer	70
	15	J	width of metal layer	100
	16	K	height of dielectric substrate	175
	17	M	width of dielectric substrate	105
	18	N	dielectric substrate thickness	1.5

**Table 3:** Antenna dimensions—final values.

	Number	Symbol	Physical Meaning	Final Value (mm)
Design variables	1	R1	radius of the smallest circle	15.3
	2	Rx2	radius of external ellipse in x direction	51.8
	3	Ry2	radius of external ellipse in y direction	73.4
	4	Rx3	radius of internal ellipse in x direction	31.8
	5	Ry3	radius of internal ellipse in y direction	39.6
	6	a	thickness of external ellipse	2.7
	7	b	thickness of internal ellipse	1.9
	8	c	internal rectangle width	6.8
	9	d	width of slot	17.6
Constant values parameters	10	e	width of interconnecting rectangle	1
	11	f	width of horizontal slot in feeding point	2
	12	g	height of metal layer at the feeding point	10
	13	h	elevation of circular part above rectangle	3
	14	i	height of metal layer	70
	15	j	width of metal layer	100
	16	k	height of dielectric substrate	175
	17	m	width of dielectric substrate	105
	18	n	dielectric substrate thickness	1.5

**Table 4:** Current distribution in optimized antenna.

Frequency (GHz)	Current Distribution Normalized to 10 A/m 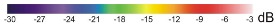
0.74	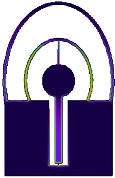
2.45	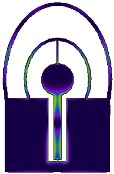
3.5	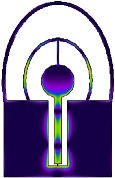
5.8	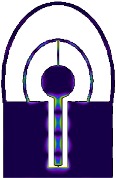

**Table 5:** Radiation patterns of optimized antenna.

Frequency (GHz)	Maximum Gain (dBi)	Gain in *z-x* plane (dBi)	Gain in *z-y* Plane (dBi)
0.74	1.3	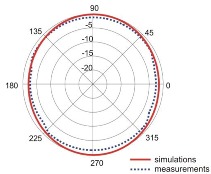	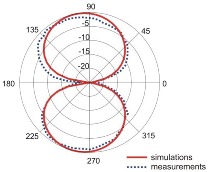
2.45	4	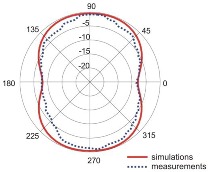	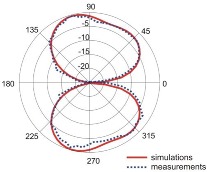
3.5	6.5	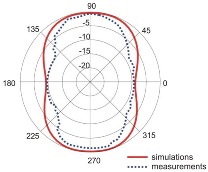	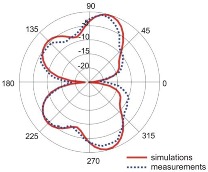
5.8	7.2